# Predicting survival rate by plasma biomarkers and clinical variables in syndromes associated with frontotemporal lobar degeneration

**DOI:** 10.1002/alz.14558

**Published:** 2025-02-12

**Authors:** Maria Sofia Cotelli, Barbara Tarantino, Kübra Tan, Hanna Huber, Valentina Cantoni, Valeria Bracca, Roberto Gasparotti, Enrico Premi, Giancarlo Logroscino, Andrea L. Benedet, Kaj Blennow, Henrik Zetterberg, Mario Grassi, Nicholas J. Ashton, Barbara Borroni

**Affiliations:** ^1^ Department of Continuity of Care and Frailty ASST Spedali Civili Brescia Italy; ^2^ Department of Brain and Behavioural Sciences Medical and Genomic Statistics Unit University of Pavia Pavia Italy; ^3^ Department of Psychiatry and Neurochemistry Institute of Neuroscience and Physiology The Sahlgrenska Academy University of Gothenburg Gothenburg Sweden; ^4^ Department of Clinical and Experimental Sciences University of Brescia Brescia Italy; ^5^ Neuroradiology Unit University of Brescia Brescia Italy; ^6^ Stroke Unit ASST Spedali Civili Brescia Italy; ^7^ Center for Neurodegenerative Diseases and the Aging Brain Pia Fondazione Cardinale Giovanni Panico University of Bari‐Aldo Moro Bari Italy; ^8^ Clinical Neurochemistry Laboratory Sahlgrenska University Hospital Gothenburg Sweden; ^9^ Paris Brain Institute ICM Pitié‐Salpêtrière Hospital, Sorbonne University Paris France; ^10^ Neurodegenerative Disorder Research Center Division of Life Sciences and Medicine and Department of Neurology Institute on Aging and Brain Disorders University of Science and Technology of China and First Affiliated Hospital of USTC Hefei P.R. China; ^11^ Department of Neurodegenerative Disease UCL Institute of Neurology London UK; ^12^ UK Dementia Research Institute at UCL London UK; ^13^ Hong Kong Center for Neurodegenerative Diseases Clear Water Bay Hong Kong China; ^14^ Wisconsin Alzheimer's Disease Research Center University of Wisconsin School of Medicine and Public Health University of Wisconsin–Madison Madison Wisconsin USA; ^15^ Banner Sun Health Research Institute Sun City Arizona USA; ^16^ Banner Alzheimer's Institute and University of Arizona Phoenix Arizona USA; ^17^ Molecular Markers Laboratory IRCCS Istituto Centro San Giovanni di Dio Fatebenefratelli Brescia Italy

**Keywords:** biological markers, corticobasal syndrome, frontotemporal dementia, frontotemporal lobar degeneration, motor neuron disease, primary progressive aphasia, progressive supranuclear palsy, survival

## Abstract

**INTRODUCTION:**

Modeling the survival rate in syndromes associated with frontotemporal lobar degeneration (FTLD) is essential to assess disease trajectories.

**METHODS:**

In 262 patients with FTLD, we considered plasma neurofilament light chain (NfL), glial fibrillary acidic protein, brain‐derived tau, phosphorylated tau217 and amyloid beta (Aβ42/Aβ40). The FTLD Survival Score (FTLD‐SS) was calculated by the β coefficients of the variables independently associated with survival rate.

**RESULTS:**

Increased plasma NfL levels (*p *< 0.001), older age at evaluation (*p *= 0.002), positive family history (*p *= 0.04), and motor phenotypes (*p *< 0.001) were associated with reduced survival. The predictive validity of FTLD‐SS was 0.75 (95% confidence interval, 0.59–0.91) at 1 year.

**DISCUSSION:**

Survival rate in FTLD is shaped by intensity of neurodegeneration (using plasma NfL as proxy) together with certain clinical variables. The FTLD‐SS may serve as a simple tool for survival rate estimation and for patient stratification in clinical trials.

**Highlights:**

- Plasma neurofilament light chain and clinical variables can predict survival in frontotemporal lobar degeneration (FTLD)–associated syndromes.
- FTLD Survival Score (FTLD‐SS), computed with survival predictors, may serve as a simple tool for patient stratification.
- FTLD‐SS is associated with greater atrophy in frontal and putamen areas.

## 1 INTRODUCTION

Frontotemporal lobar degeneration (FTLD) encompasses a heterogeneous group of neurodegenerative disorders with a wide range of clinical, genetic, and neuropathological features, and the course of disease remains poorly predictable.^1^ Behavioral and personality changes characterize the behavioral variant frontotemporal dementia (bvFTD),^2^ while speech and language deficits are prominent symptoms of primary progressive aphasia (PPA).^3^ A proportion of patients have associated extrapyramidal symptoms which characterize either progressive supranuclear palsy (PSP)^4^ or corticobasal syndrome (CBS),^5^ while others present with overlapping motor neuron disease which defines frontotemporal dementia amyotrophic lateral sclerosis (FTD‐ALS).^6^

In recent years, our understanding of the biological and clinical bases of FTLD‐associated syndromes has progressively improved.^7^, ^8^ However, there is an urgent need for better prognostic models to aid both trial design and clinical management. Knowledge of the survivorship trajectories and related predictors is critical to assess the efficacy of treatment interventions and to promote adequate planning of public health service policies.

In this context, the available literature on FTLD survival rate is still limited and often based on small clinical series without considering the entire FTLD spectrum.^9^, ^10^, ^11^, ^12^, ^13^, ^14^, ^15^, ^16^ It has been clearly demonstrated that the FTD‐ALS phenotype is associated with poorer prognosis,^11^, ^17^ and more recently extrapyramidal symptoms have been related to shorter survival.^15^, ^17^ Moreover, it has been suggested that FTLD with genetic traits, due to pathogenetic mutations or familial disease, may present worse prognosis.^18^, ^19^, ^20^

Emerging plasma biological markers of neurodegenerative disorders may play a role in estimating FTLD survival probability, ^21^, ^22^ but studies comprehensively evaluating their performance in predicting survivorship are not yet available. Plasma biological markers are cost effective, easy to perform, and widely available, so it is of great interest to determine their potential contribution.

Neurofilament light chain (NfL) and glial fibrillary acidic protein (GFAP) are indicative of neurodegenerative processes and astroglial activation, respectively, and have been related to disease progression in FTLD‐associated syndromes.^21^, ^22^ Plasma brain‐derived tau (BD‐tau), a marker for Alzheimer's disease (AD) and neuronal and axonal damage,^23^ showed good performance in predicting outcomes in stroke and traumatic brain injury,^24^, ^25^ suggesting a plausible importance in prognosis prediction in neurological disorders. Finally, it might be hypothesized that AD co‐pathology, as measured by phosphorylated tau (p‐tau)217 or amyloid beta (Aβ)42/Aβ40 ratio,^26^, ^27^ might affect survival rate in FTLD.

Given these premises, in a large cohort of patients with FTLD‐associated syndromes, we aimed to (1) assess the ability of plasma biological markers, such as NfL, GFAP, BD‐tau, p‐tau217, and Aβ42/Aβ40 ratio, to predict survival probability; (2) develop a survival probability risk score at the individual patient level, considering identified biological markers and clinical variables; and (3) determine which are the brain correlates of the developed survival probability risk score.

## 2 METHODS

### 2.1 Study population

This retrospective study included 262 consecutive patients with FTLD‐associated syndromes recruited at the Centre for Neurodegenerative Disorders, University of Brescia, Italy, for whom plasma biomarkers were available.

The patients included in this study met current clinical criteria for the diagnosis of bvFTD, semantic and non‐fluent variants of PPA (svPPA and nfvPPA), FTD‐ALS, PSP, or CBS^.^ ^2^, ^3^, ^4^, ^5^, ^6^

Each FTLD patient underwent a neurological evaluation, routine laboratory examination, and a neuropsychological and behavioral assessment.

Demographic and clinical characteristics were carefully recorded. Family history was assessed according to the modified Goldman score (GS), where GS = 1 is an autosomal dominant family history of FTLD‐associated syndromes or ALS, GS = 2 is familial aggregation of ≥ 3 family members with dementia but not meeting criteria for 1, GS = 3 is one other affected family member with dementia, and GS = 4 is no or unknown family history.^28^ Disease severity at time of diagnosis was measured by the Clinical Dementia Rating (CDR) dementia staging instrument plus behavior and language domains from the National Alzheimer's Coordinating Center and frontotemporal lobar degeneration modules (CDR plus NACC) rating scale.

In all cases, the diagnosis was supported by brain structural imaging, while cerebrospinal fluid (CSF) concentrations of tau, p‐tau_181_, and Aβ_1‐42_ or positron emission tomography (PET) amyloid scan were measured in a subset of cases to rule out AD, as previously reported.^20^ Furthermore, in familial cases (based on the presence of at least one dementia case among the first‐degree relatives) and early onset sporadic cases, genetic screening for monogenic causes of FTD was carried out.

Each participant underwent blood collection for the measurements of plasma biomarkers at the time of evaluation, and a subset of patients underwent standardized brain magnetic resonance imaging (MRI) at the time of evaluation (*n* = 111) to evaluate the correlation between brain atrophy and predictors of survival.

By April 1, 2024, each patient was re‐evaluated by in‐person examination by the referral team or the patient's status was ascertained via telephone call with the caregiver or by inquiries to administrative electronic health records; in case of previous death of any cause, the exact date was recorded.

The study was compliant with the Standards of Reporting of Neurological Disorders (STROND) for observational studies.^29^ Full written informed consent was obtained from all participants or their legal representatives in accordance with the Declaration of Helsinki and the study was approved by the Brescia Hospital Ethics Committee (NP2189).

**RESEARCH IN CONTEXT**

1. **Systematic review**: Studies estimating survival rate and its predictors in syndromes associated with frontotemporal lobar degeneration (FTLD) are still limited. Plasma biomarkers for neurodegenerative disorders may improve survival prediction.
2. **Interpretation**: Increased plasma neurofilament light chain levels, older age at evaluation, positive family history, and motor phenotypes predicted reduced survival. A point‐scoring system, the FTLD Survival Score (FTLD‐SS), was calculated at single‐subject level.
3. **Future directions**: The FTLD‐SS may serve as a simple tool for survival rate estimation and for patient stratification in clinical trials. Future confirmatory studies are needed.

### 2.2 Plasma biomarkers assessment

Plasma samples were collected according to standard procedures and stored at −80°C until use. Biological analyses were carried out at the University of Gothenburg, Sweden. Plasma NfL, GFAP, Aβ40, and Aβ42 were quantified using the commercial Neurology 4‐plex E kit (103670; Quanterix), as previously published.^27^ Plasma p‐tau217 was quantified using the ALZpath Quanterix^27^ and BD‐tau was measured using validated in‐house assays.^23^ Samples were run in duplicate on a Quanterix Simoa‐HD‐X platform, with intra‐ and inter‐assay variation < 15% for all biomarkers.

### 2.3 MRI acquisition, pre‐processing, and analysis

Brain structural images (three‐dimensional T1‐weighted magnetization‐prepared rapid acquisition with gradient echo [MPRAGE] MRI) were collected (Siemens Skyra 3T). The raw Digital Imaging in Communications in Medicine scans were converted into the Neuroimaging Informatics Technology Initiative (NIfTI) format using the MRIcroGL software (www.nitrc.org/projects/mricrogl). T1‐weighted images were then processed and analyzed with the voxel‐based morphometry (VBM) pipeline implemented in the Computational Anatomy Toolbox (CAT12 v.1742; www.neuro.uni‐jena.de/cat) for Statistical Parametric Mapping (SPM12, v.7219; www.fil.ion.ucl.ac.uk/spm/software/spm12) running on MATLAB R2019b (the MathWorks, Inc.). The VBM pipeline consists of several stages (tissue segmentation, spatial normalization to a standard Montreal National Institute [MNI] template, modulation, and smoothing), as previously described.^30^ CAT12 potentially provided a more robust and accurate performance compared to other VBM pipelines^31^ in the calculation of gray matter volume (GMV). The normalized and modulated gray matter images were then smoothed with 8 × 8 × 8 mm^3^ full width at half‐maximum Gaussian kernel.

A regression analysis was performed in SPM12 evaluating the association with the generated survival score. Sex and total intracranial volume (TIV = GMV + white matter volume + CSF volume) were used as nuisance variables in the model. A statistical threshold of *p *< 0.001 uncorrected was adopted, with a minimum cluster size of 700 voxels.

### 2.4 Statistical analysis

Continuous and categorical variables are reported as mean (standard deviation) and % (numbers), respectively. Demographic and clinical variables were compared using one‐way analysis of variance or chi‐square test, as appropriate.

Survival was calculated as time from evaluation to time of death from any cause (outcome = 1) or censoring date (April 1, 2024, outcome = 0).

The Kaplan–Meier survival analysis was used to estimate the cumulative incidence of outcome events by follow‐up time. Hazard ratios (HRs) and 95% confidence intervals (CIs) were assessed by Cox proportional hazards models in univariable analyses to examine the effect of demographic, clinical, and biological variables on survival rate and to detect the independent predictors of outcome.

We assessed the proportional hazards assumption for a Cox regression model fit by plotting estimates of the time‐independent coefficient *β* versus time. If the proportional hazards assumption is true, each *β* is a horizontal line.

We developed the FTLD Survival Score (FTLD‐SS) considering possible predictors of poor outcome. To select predictors, we used the least absolute shrinkage and selection operator (LASSO) method proposed by Tibshirani for survival analysis.^32^ This is a penalized variable selection technique, which shrinks *β* coefficients (*β* = ln[HR]) and produces some *β* coefficients that are exactly zero. The variables whose *β* coefficient is zero are then automatically deleted from the predictor set. Model screening was performed by tuning penalized parameters with *K*‐fold cross‐validation,^33^ with *K* = 10 and approximately equal‐sized subsets. The non‐zero *β* coefficients of each predictor variable from the multivariable survival model with minimum LASSO penalty were used to generate a weighted scoring system of the predictors. An overall continuous individual FTLD‐SS for each patient (1) was calculated by summing up each *β* coefficient × each predictor value (*s[i*] = Σ*j β*[*j] × [ij*]). The exponential of FTLD‐SS, *η*(*i*) = exp(*s[i*]), represents the hazard score for each subject. Higher values of *η*(*i*) correspond to a higher hazard level and a shorter survival time based on the predictors. To assess the predictive validity of the FTLD‐SS, we used the receiver operating characteristic curves, the area under the receiver operating characteristic curve (AUC), and the discrimination C‐statistic (overall AUC), which consider the timing of events from survival data (at 1, 3, and 5 years for AUC and from 0 to 5 years for C‐statistic).^34^

AUC and C summaries are 0‐to‐1 values, where 50% is the null value of worst‐case scenario for decision making. To account for the fact that we evaluated the risk score function on the same data on which it was developed, the C‐statistic (overall AUC) in predicting events that occur in a time range 0 to *t* was validated by *K*‐fold cross‐validation with *K* = 10, each fold evaluating a test sample by using scores obtained from the *β* coefficients trained by the other learning sample. In this way, we corrected for potential overfitting in the assessment of the score performance. The optimal cut‐off of the FTLD‐SS was defined according to the Youden index method.^35^ The cut‐off was used to convert the FTLD‐SS into binary data and assess the Kaplan–Meier survival curves between groups.

Two‐sided values of *p *< 0.05 were considered significant. Statistical analyses were conducted with the software R (version 4.4.1 R Development Core Team, 2024).

## 3 RESULTS

### 3.1 Population

Two hundred sixty‐two patients with FTLD‐associated syndromes were included in the study. The cohort consisted of 127 bvFTD, 86 PPA (62 nfvPPA and 24 svPPA), 39 PSP/CBS (17 PSP and 22 CBS), and 10 FTD‐ALS patients.

The demographic and clinical characteristics of patients with FTLD‐associated syndromes are reported in Table 1.

**TABLE 1:** Demographic and clinical characteristics of incident frontotemporal lobar degeneration patients.

	bvFTD	PPA^a^	PSP/CBS^b^	FTD‐ALS	FTLD (all)	
*Variables*	*N* = 127	*N* = 86	*N* = 39	*N* = 10	*N* = 262	*p* value^*^
***Demographics and clinical***						
Age at evaluation, years	64.4 (9.0)	65.4 (8.8)	67.2 (6.4)	63.0 (6.3)	65.0 (8.5)	0.27
Sex, female % (*n*)	33.9 (43)	52.3 (45)	43.6 (17)	1 (10)	40.5 (106)	0.01^**^
Age at onset, years	60.6 (8.8)	62.6 (9.0)	63.4 (6.8)	59.2 (6.0)	61.7 (8.5)	0.20
Education, years	10.2 (4.5)	11.5 (4.0)	8.7 (3.5)	11.7 (3.8)	10.5 (3.8)	0.04
Goldman score, ≤ 2% (*n*)^c^	37.8 (45)	30.9 (25)	19.4 (6)	70.0 (7)	34.4 (83)	0.02
CDR plus NACC	1.3 (0.8)	1.0 (0.7)	0.9 (0.7)	1.0 (0.5)	1.2 (0.8)	0.06
Death, % (*n*)	32.3 (41)	32.6 (28)	56.4 (22)	63.2 (43)	90 (9)	<0.01^**^
***Plasma biomarkers***						
NfL (pg/mL)	55.7 (43.7)	76.5 (47.4)	61.6 (79.8)	126.9 (90.2)	66.2 (55.7)	<0.001
GFAP (pg/mL)	227.6 (130.5)	300.0 (164.7)	234.8 (109.6)	204.0 (83.8)	251.6 (142.3)	0.001
BD‐tau (pg/mL)	0.92 (0.70)	1.01 (0.82)	0.71 (0.62)	0.97 (0.61)	0.92 (0.73)	0.218
p‐tau217 (pg/mL)	0.33 (0.40)	0.45 (0.77)	0.35 (0.46)	0.50 (0.38)	0.38 (0.55)	0.421
Aβ42/Aβ40	0.08 (0.05)	0.08 (0.05)	0.09 (0.07)	0.10 (0.03)	0.08 (0.05)	0.764

*Note*: Results are expressed as mean (standard deviation), unless otherwise specified.

Abbreviations: Aβ, amyloid beta; BD‐tau, brain‐derived tau; bvFTD, behavioral variant frontotemporal dementia; CBS, corticobasal syndrome; CDR plus NACC, Clinical Dementia Rating Dementia Staging Instrument plus behavior and language domains from the National Alzheimer's Coordinating Center and Frontotemporal lobar degeneration modules rating scale; FTD‐ALS, frontotemporal dementia with amyotrophic lateral sclerosis; GFAP, glial fibrillary acidic protein; *n*, number; NfL, neurofilament light chain; PPA, primary progressive aphasia; PSP, progressive supranuclear palsy; p‐tau, phosphorylated tau.

^a^ PPA: 62 non‐fluent variant PPA; 24 semantic variant PPA.

^b^ PSP/CBS: 17 PSP; 22 CBS.

^c^ Missing values: Goldman score (*n* = 21).

* One‐way analysis of variance, unless otherwise specified;

** chi‐square test.

### 3.2 Survival in FTLD

As of the census day, 100 patients (38.2%) had died, and the median survival for participants with FTLD‐associated syndromes was 1056 days (range = 59–4917 days) from time of evaluation.

All fitted models met the Cox proportional hazard assumption. Multivariable regression indicated poorer prognosis (*p *< 0.05) according to higher plasma NfL levels (HR [95% CIs] = 1.01 [1.01–1.01]), older age at evaluation (1.11 [1.04–1.19]), higher Goldman score (0.87 [0.76–0.99]), and FTD‐ALS or PSP/CBS phenotypes (2.72 [1.64–4.49]; see Table 2). There were no differences in HRs according to sex or education; GFAP and markers of AD co‐pathology, that is, BD‐tau, p‐tau217, and Aβ42/Aβ40 ratio, were not found to have a significant effect on survival rate.

**TABLE 2:** Cox proportional hazard models of overall survival considering phenotype, demographic and clinical variables, and biomarkers.

	Univariate analysis		Multivariate analysis	
	HR (95% CI)	*p* value	HR (95% CI)	*p* value
***Plasma biomarkers***				
NfL (pg/mL)	1.01 (1.01–1.01)	<0.001	1.01 (1.01–1.01)	<0.001
GFAP (pg/mL)	1.00 (1.00–1.00)	0.92	1.00 (1.00–1.00)	0.92
BD‐tau (pg/mL)	1.40 (0.96–2.05)	0.08	1.26 (0.75–2.13)	0.38
p‐tau217 (pg/mL)	1.14 (0.75–1.73)	0.05	0.97(0.50–1.88)	0.92
Aβ42/Aβ40	1.50 (0.08–28.1)	0.79	1.57 (0.09–26.6)	0.75
***Demographic and clinical variables***				
Age at diagnosis, years	1.06 (1.03–1.09)	< 0.001	1.11 (1.04–1.19)	0.003
Sex, female	0.97 (0.65–1.45)	0.87	0.67 (0.41–1.10)	0.11
Education, years	0.98 (0.94–1.02)	0.74	1.01 (0.96–1.06)	0.74
Goldman score, 1–4	0.93 (0.83–1.04)	0.20	0.87 (0.76–0.99)	0.04
FTD‐ALS or PSP/CBS vs. bvFTD	2.00 (1.30–3.06–)	<0.001	2.72 (1.64–4.49)	<0.001
PPA vs bvFTD	1.01 (0.65–1.57)	0.95	1.12 (0.65–1.93)	0.67

Abbreviations: Aβ, amyloid beta; BD‐tau, brain‐derived tau; bvFTD, behavioral variant frontotemporal dementia; CBS, corticobasal syndrome; CI, confidence interval; FTD‐ALS, frontotemporal dementia with amyotrophic lateral sclerosis; GFAP, glial fibrillary acidic protein; GS, Goldman score; HR, hazard ratio; NfL, neurofilament light chain; PPA, primary progressive aphasias; PSP, progressive supranuclear palsy; p‐tau, phosphorylated tau.

### 3.3 FTLD‐SS

We used the LASSO technique for variable selection, using biological markers and demographic and clinical variables as reported in Table 2 and Figure S1 in supporting information. This confirmed the non‐zero *β* coefficients of plasma NfL, age at evaluation, GS, and FTD‐ALS or PSP/CBS phenotypes.

The FTLD‐SS was generated using the weighted *β* coefficients of these independent predictor variables after (0,1) recoding, as follows: 1 = age > 65 years, 1 = NfL > 66 pg/mL, 1 = GS < 3, and 1 = FTD‐ALS or PSP/CBS phenotype (see Table 3). The sum of the weighted scores × binary variables was used to estimate the overall score. This gave a continuous score whose values range between 0 and 3.

**TABLE 3:** FTLD‐SS (frontotemporal lobar degeneration survival score) predictors selected by the LASSO (least absolute shrinkage and selection operator) procedure.

Variable	HR (95% CI)	*p* value	Score points
Age at evaluation, > 65 years	1.91 (1.28–2.87)	0.002	0.6
Goldman score, ≤ 2	1.58 (1.02–2.45)	0.04	0.5
Plasma NfL, > 66 pg/mL	2.36 (1.51–3.69)	<0.001	0.9
Phenotype, FTD‐ALS or PSP/CBS	2.80 (1.74–4.48)	<0.01	1.0

Abbreviations: CBS, corticobasal syndrome; FTD‐ALS, frontotemporal dementia with amyotrophic lateral sclerosis; HR, hazard ratio; NfL, neurofilament light chain; PSP, progressive supranuclear palsy.

The individual risk over 1, 3, and 5 years can be calculated using the following formula:

1‐year risk = 1−0.99^exp(FTLD‐SS)^; 3‐year risk = 1−0.92^exp(FTLD‐SS)^, 5‐year risk = 1−0.79^exp(FTLD‐SS)^.

The FTLD‐SS score offered moderate discrimination with an AUC of 0.75 (95% CI, 0.59–0.91) at 1 year, 0.73 (95% CI, 0.65–0.82) at 3 years, and 0.66 (95% CI, 0.54–0.78) at 5 years, indicating an acceptable predictive performance of our model. Mean 10‐fold cross‐validated C‐statistics were 0.74, 0.73, and 0.66 from 0 to 1 year, 3 years, and 5 years, respectively, suggesting that the bias resulting from predicting on the same data set used for fitting was ≈ 0.1 (see Figure S2 in supporting information).

**FIGURE 1:**
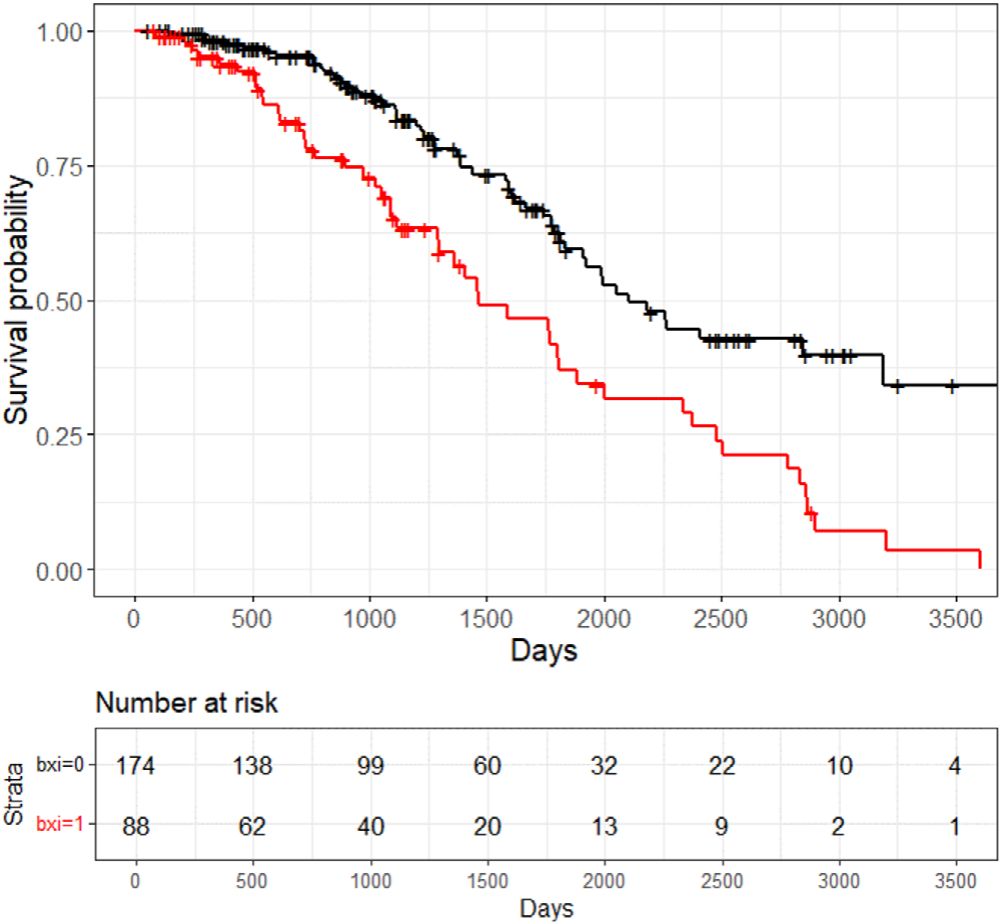
Survival probability in syndromes associated with FTLD patients according to FTLD‐SS. bx = 0 FTLD‐SS ≤ 1.3 (black line). bx = 1 FTLD‐SS > 1.3 (red line). FTLD, frontotemporal lobar degeneration; FTLD‐SS, FTLD Survival Score

Figure 1 shows the Kaplan–Meier survival curves for the two groups classified by the optimal FTLD‐SS cut‐off of 1.32, based on the Youden index at 1 year, with sensitivity = 66% and specificity = 74%.

**FIGURE 2:**
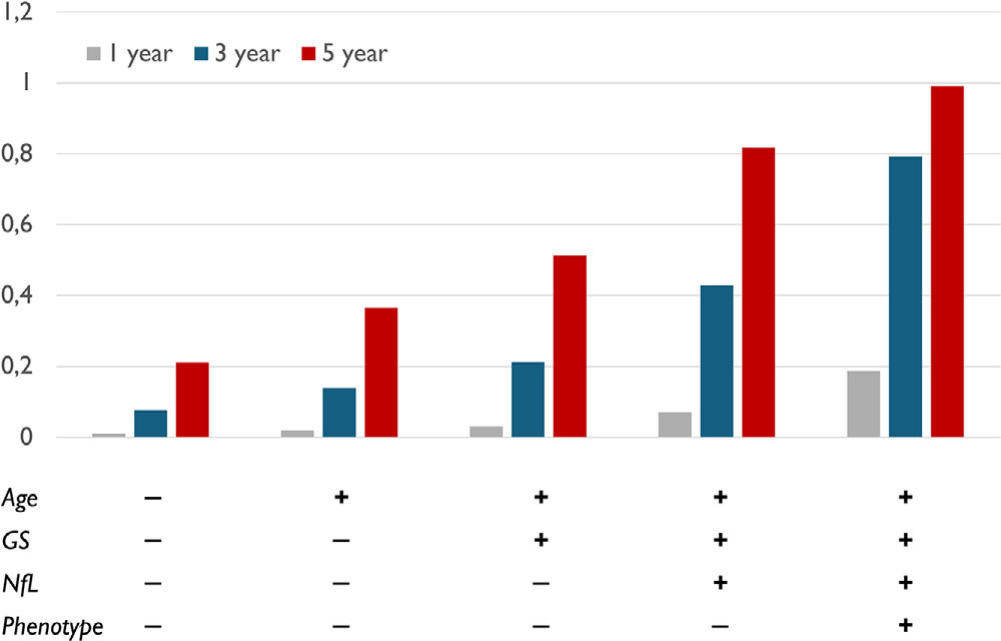
One‐year, 3‐year, and 5‐year estimated risk considering independent predictors. Independent predictors: age (− ≤ 64 years; + > 65 years); GS = Goldman score (−GS = 3 or GS = 4; +GS = 1 or GS = 2); neurofilament light chain (NfL; − ≤ 65; + > 66); phenotype (−, behavioral variant frontotemporal dementia or primary progressive aphasia; +, frontotemporal dementia with amyotrophic lateral sclerosis or progressive supranuclear palsy or corticobasal syndrome).

In Figure 2, we report the estimated 1‐year, 3‐year, and 5‐year mortality risk in FTLD patients with varied combinations of predictors: age (− ≤ 64 years; + > 65 years); GS (− = 3 or 4; + = 1 or 2), plasma NfL (− ≤ 65; + > 66), phenotype (− bvFTD or PPA; + FTD‐ALS or PSP or CBS). For each combination, the 3‐year and 5‐year models give risk estimates that are 4 to 5 times higher than those of the 1‐year model. In particular, the 5‐year estimated risk of shorter survival was clearly affected by plasma NfL and clinical phenotype. For example, the 1‐year risk for a patient with all four positive predictors is ≈ 20%, but the corresponding 3‐year risk reaches ≈ 80% and the 5‐year risk reaches ≈ 100% (see Table S1 in supporting information).

### 3.4 Imaging correlates of FTLD‐SS

An MRI suitable for voxel‐based morphometry analyses was available in 42% (111/262) of patients (51 bvFTD, 40 PPA, 14 PSP/CBS, 6 FTD‐ALS).

**FIGURE 3:**
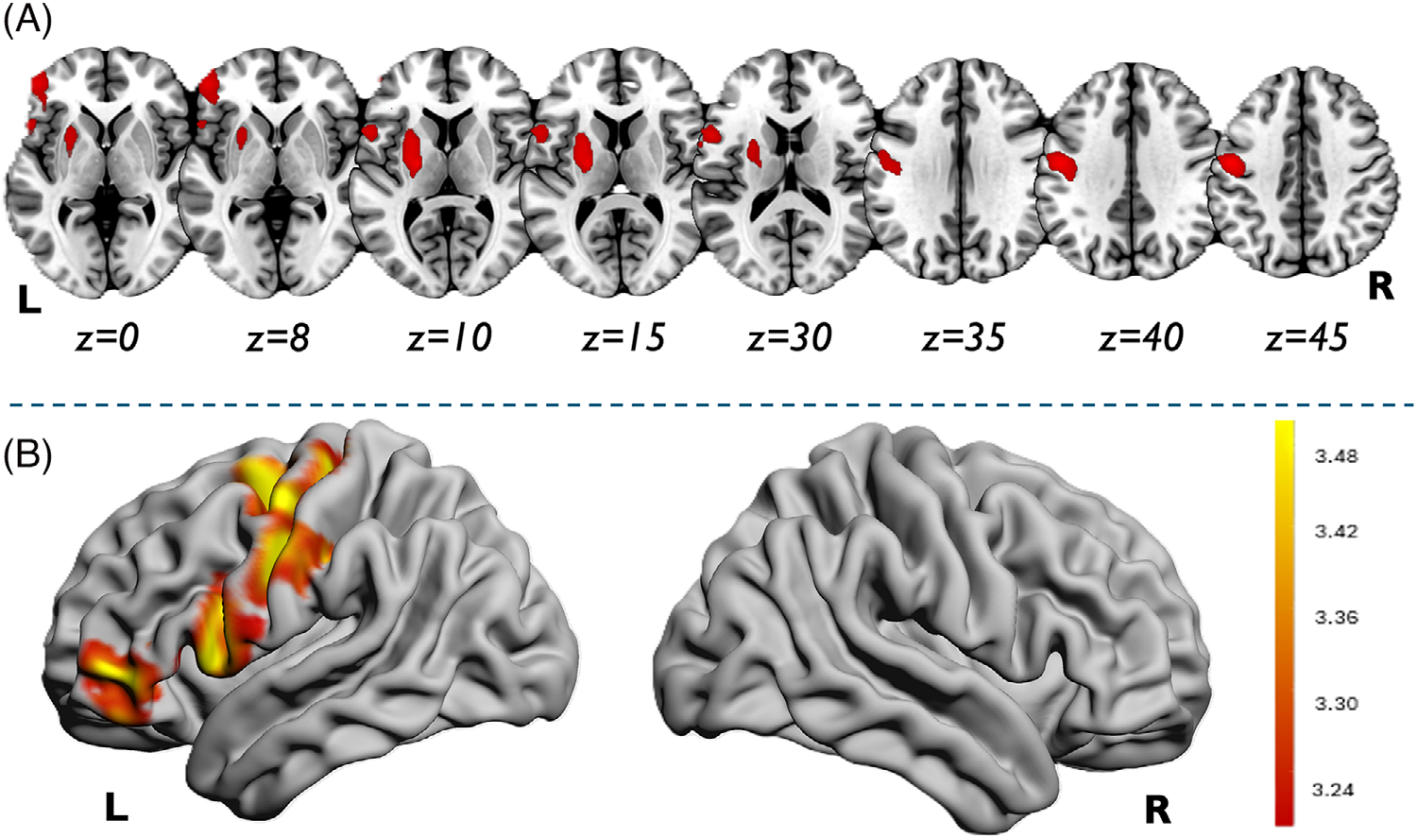
FTLD‐SS correlation with regional brain atrophy. A, Correlation analysis between higher FTLD‐SS and greater brain atrophy (*p *< 0.001 uncorrected). Results were superimposed on 2D standard MNI template. B, Representative 3D rendering of the correlation analyses results (Surf Ice toolbox: https://www.nitrc.org/projects/surfice/) (*p *< 0.001 uncorrected). L = left, R = right. FTLD‐SS, frontotemporal lobar degeneration Survival Score; MNI, Montreal National Institute.

As reported in Figure 3, FTLD‐SS was correlated with gray matter density (as a proxy of brain atrophy) in the left precentral gyrus (*x *= −50, *y *= −9, *z *= 38; *T *= 4.51, cumulative cluster of 2362 voxels), in the left putamen (*x *= −22, *y *= −10, *z *= 9; *T *= 4.27, cumulative cluster of 1288 voxels), in the left inferior frontal gyrus (*x *= −52, *y *= 46, *z *= −16; *T *= 4.27, cumulative cluster of 1440 voxels), and in the left middle frontal gyrus (*x *= −28, *y *= −2, *z *= 62; *T *= 4.21, cumulative cluster of 1868 voxels), all *p *< 0.001 uncorrected. The opposite comparison did not show any clusters surviving the pre‐established thresholds.

## 4 DISCUSSION

Modeling survival in the syndromes associated with FTLD is crucial to assess natural disease trajectories and stratify patients according to different progression in future intervention trials.

In the present study, we evaluated the role of wide set of plasma biomarkers along with clinical variables, and we developed the FTLD‐SS. We found that increased plasma NfL levels, older age at evaluation, motor phenotype, and positive family history were associated with reduced survival. Based on these variables, the FTLD‐SS was able to predict prognosis at individual subject level with accuracy of ≈ 80%.

The present findings confirm and extend previous literature reporting blood NfL as a reliable tool to predict survival rate in FTLD,^21^, ^22^ ALS,^36^ and in α‐synucleopathies.^37^ We did not find any significant contribution of other biological markers in predicting survivorship in syndromes associated with FTLD. The few previous works on the role of GFAP in FTLD showed conflicting results,^22^, ^38^ and even in the present study we did not find a significant association between plasma GFAP levels and survival rate. We also argue that AD‐related biomarkers, such as BD‐tau, p‐tau217, or Aβ42/Aβ40 ratio,^26^, ^27^ do not influence survival rate in syndromes associated with FTLD. Indeed, additional peripheral inflammatory markers, recently related to survival probability in FTLD, or new advanced approaches to assess multiple markers such as NUcleic acid Linked Immuno‐Sandwich Assay (NULISA), should be considered to further shape FTLD‐SS and increase its accuracy.^39^, ^40^

Along with plasma NfL levels, clinical phenotype, age at diagnosis, and GS contributed to FTLD‐SS. As in previous studies, we confirm that features of motor neuron disease worsen the prognosis in FTLD,^11^, ^12^ and older age is an independent predictor of mortality.^14^ However, survival rate also depended on the presence of phenotypes characterized by extrapyramidal signs (i.e., PSP and CBS), confirming recent findings.^15^, ^17^ Poorer prognosis in PSP and CBS may relate to an increased risk of complications (e.g., falls, fracture) and frailty, and in turn mortality.^15^ Finally, familial trait, as measured with the GS,^28^ was significantly associated with poorer prognosis, in particular in patients with either autosomal dominant family history or familial aggregation of three or more family members with dementia. This is in line with the evidence that genetic FTD may present more aggressive disorder.^18^, ^19^, ^20^

These independent predictors allowed us to compute the FTLD‐SS, which provided acceptable accuracy in estimating survival probability. In this view, FTLD‐SS may be of help in designing future pharmacological and non‐pharmacological trials, considering patient inclusion according to survival rate risk and trial length.

Interestingly, higher FTLD‐SS was associated with greater brain atrophy and significantly correlated with brain areas of the dominant hemisphere, such as the precentral area, the putamen, and the middle‐inferior frontal gyrus. Notably, the higher the FTLD‐SS, the greater the atrophy in motor areas, and in areas primarily involved in the disease. These areas are also related to clinical phenotype primarily included in FTLD‐SS.

We acknowledge that, while the findings of the present study are promising, there are several limitations that should be acknowledged. One significant limitation is the lack of autopsy‐proven diagnoses, which is considered the gold standard for confirming neurodegenerative diseases. However, the majority of participants did undergo CSF analysis or amyloid PET imaging, and plasma levels of p‐tau217 and Aβ42/Aβ40 ratio herein reported further excluded AD. Second, the monocentric design may limit the generalizability of the findings, which could be improved by multi‐center studies and considering additional biomarkers and clinical variables to further model the FTLD‐SS.

Despite these limitations, this is the first study on survival in syndromes associated with FTLD in such a large panel of biomarkers that were concomitantly assessed, and a large cohort encompassing the range of clinical FTLD phenotypes evaluated. Moreover, we considered survival rate as outcome of prognostic utility, as the high clinical heterogeneity across the FTLD spectrum and measurement ceiling effects make it difficult to capture clinical progression with cognitive or behavioral tests.^40^

In conclusion, plasma NfL along with clinical variables may be of help in predicting survival in clinical syndromes associated with FTLD and may be used to improve patient stratification in future trials and contribute to the appropriate planning of public health service policies.

## CONFLICT OF INTEREST STATEMENT

H.Z. has served on scientific advisory boards and/or as a consultant for Abbvie, Acumen, Alector, Alzinova, ALZpath, Amylyx, Annexon, Apellis, Artery Therapeutics, AZTherapies, Cognito Therapeutics, CogRx, Denali, Eisai, LabCorp, Merry Life, Nervgen, Novo Nordisk, Optoceutics, Passage Bio, Pinteon Therapeutics, Prothena, Red Abbey Labs, reMYND, Roche, Samumed, Siemens Healthineers, Triplet Therapeutics, and Wave, has given lectures sponsored by Alzecure, BioArctic, Biogen, Cellectricon, Fujirebio, Lilly, Novo Nordisk, Roche, and WebMD, and is a co‐founder of Brain Biomarker Solutions in Gothenburg AB (BBS), which is a part of the GU Ventures Incubator Program (outside submitted work). K.B. has served as a consultant and on advisory boards for Abbvie, AC Immune, ALZPath, AriBio, BioArctic, Biogen, Eisai, Lilly, Moleac Pte. Ltd, Neurimmune, Novartis, Ono Pharma, Prothena, Roche Diagnostics, Sanofi, and Siemens Healthineers; has served on data monitoring committees for Julius Clinical and Novartis; has given lectures, produced educational materials, and participated in educational programs for AC Immune, Biogen, Celdara Medical, Eisai, and Roche Diagnostics; and is a co‐founder of Brain Biomarker Solutions in Gothenburg AB (BBS), which is a part of the GU Ventures Incubator Program, outside the work presented in this paper. B.B. has served on scientific advisory boards for Alector, Alexion/Astrazeneca, AviadoBio, Lilly, Denali, Wave, UCB. The other authors do not have conflicts of interest. Author disclosures are available in the supporting information.

## Supporting information



Supporting Information

Supporting Information

## Data Availability

All study data, including anonymized raw and analyzed data, and materials will be available from the corresponding author upon reasonable request, under the condition that the recipients do not attempt to de‐anonymize data nor pass them on third parties.
