# Supplementary material for: Predicting survival rate by plasma biomarkers and clinical variables in syndromes associated with frontotemporal lobar degeneration
Source: Alzheimers Dement. 2025 Feb 12;21(2):e14558. doi: 10.1002/alz.14558 (PMC11815202; doi:10.1002/alz.14558)
Supplement: Supplementary file 2 — Supporting Information [file ALZ-21-e14558-s002.docx]

**Supplementary Materials**

**Supplementary Table 1.** One-year, 3-year and 5-year estimated risk considering independent predictors.

| **Predictors** | Age (-)  GS (-)  Nfl (-)  Phenotype (-) | Age (+)  GS (-)  Nfl (-)  Phenotype (-) | Age (+)  GS (+)  Nfl (-)  Phenotype (-) | Age (+)  GS (+)  Nfl (+)  Phenotype (-) | Age (+)  Nfl (+)  GS (+)  Phenotype (+) |
| --- | --- | --- | --- | --- | --- |
| 1-year | 1.03% | 2.0% | 3.1% | 7.1% | 18.7% |
| 3-year | 7.5% | 14.0% | 21.1% | 42.9% | 79.1% |
| 5-year | 21.1% | 36.5% | 51.2% | 81.6% | 99.1% |

Independent predictors: age (−, <= 64 years; +, >65 years); GS = Goldman’s score (−, GS = 3 or GS = 4; +, GS = 1 or GS = 2); NfL = plasma neurofilament light (−, <=65; +, >66); Phenotype (−, behavioural variant Frontotemporal Dementia or Primary Progressive Aphasia; +, Frontotemporal Dementia with Amyotrophic Lateral Sclerosis or Progressive Supranuclear Palsy or Corticobasal Syndrome).

# Supplementary Figure 1. Least absolute shrinkage and selection operator (LASSO) regression was used to reduce the dimension of the grouping characteristics.


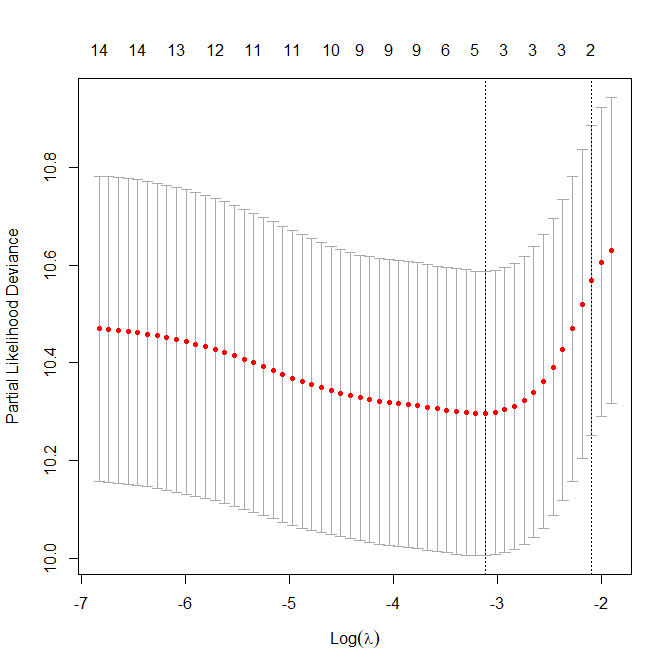


**Supplementary Figure 2.** **Predictive performance of the FTLD-SS (Frontotemporal Lobar Degeneration Survival Score).**


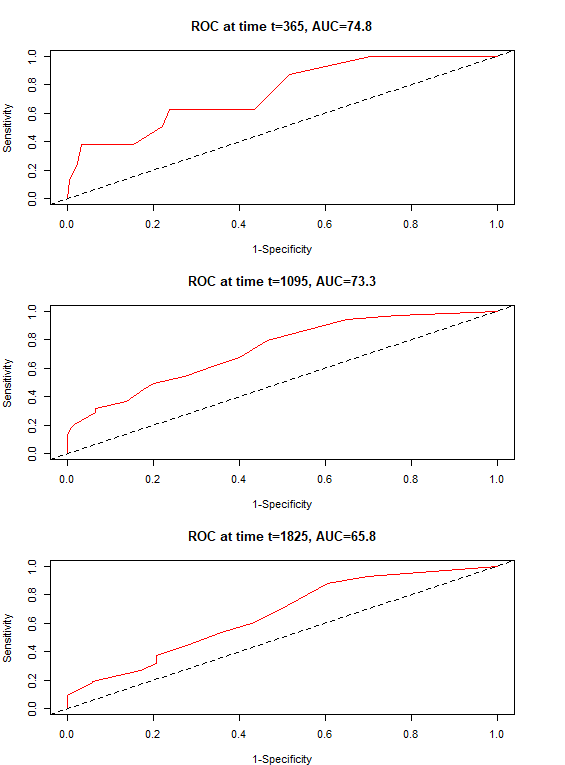


**Receiver Operating Characteristic (ROC) curves to obtain the C statistic for survival rate at 1 year, 3 years and 5 years. AUC indicates area under the ROC curve; FP = false positive; TP = true positive.**
